# The Potential Mechanisms of Cinobufotalin Treating Colon Adenocarcinoma by Network Pharmacology

**DOI:** 10.3389/fphar.2022.934729

**Published:** 2022-06-23

**Authors:** Jiyan Wang, Hongkai Chang, Meng Su, Huifang Zhao, Yaya Qiao, Yu Wang, Luqing Shang, Changliang Shan, Shuai Zhang

**Affiliations:** ^1^ State Key Laboratory of Medicinal Chemical Biology, College of Pharmacy and Tianjin Key Laboratory of Molecular Drug Research, Nankai University, Tianjin, China; ^2^ School of Life Science and Bio-Pharmaceutics, Shenyang Pharmaceutical University, Shenyang, China; ^3^ School of Integrative Medicine, Tianjin University of Traditional Chinese Medicine, Tianjin, China

**Keywords:** network pharmacology, cinobufotalin, colon cancer, signal transduction, apoptosis

## Abstract

Network pharmacology, as a novel way using bioinformatics to explore drug targets and interactions in cancer, broadens our understanding of drug action, thereby facilitating drug discovery. Here, we utilized network pharmacology to explore the role and mechanism by which cinobufotalin functions in colon adenocarcinoma (COAD). We found that cinobufotalin represses the growth and proliferation of colon cancer cells, and integrated public databases for targets reported to be associated with COAD, together with those predicted to be targets of cinobufotalin. Targets overlapped between COAD-associated proteins and cinobufotalin target proteins were used to filter candidate targets of cinobufotalin in COAD. The following proteins were thought to occupy a key position in COAD-cinobufotalin target networks: SRC, PIK3R1, MAPK1, PIK3CA, HSP90AA1, CTNNB1, GRB2, RHO1, PTPN11, and EGFR. The networks regulated by cinobufotalin were involved mainly in extracellular signal stimulation and transduction, including MAPK signaling pathway, PI3K-AKT signaling pathway, and JAK-STAT signaling pathway. Besides, transcriptome sequencing results also indicated that cinobufotalin inhibits the response of colon cancer cells to extracellular stimulation and promotes cell apoptosis. Molecular docking results showed that cinobufotalin matches in the pocket of the top candidate cinobufotalin target proteins (SRC, PIK3R1, MAPK1 and PIK3CA). These findings demonstrate cinobufotalin can be developed as potential anti-cancer therapeutics.

## Introduction

Colon adenocarcinoma (COAD) is the most common diagnosis and deadliest malignancy (Recio-Boiles and Cagir, 2022). The high incidence rate of COAD continues to increase markedly worldwide (Mork et al., 2015). COAD is mainly treated by surgical resection. Various target drugs and chemotherapy drugs have also been developed, however, the cure rate and postoperative survival quality of patients with COAD have not improved significantly (Roh et al., 2009). Therefore, novel therapeutic strategies need to be identified and developed to improve the treatment outcomes of patients with COAD.

Our previous studies demonstrated that the drug combination therapy has a significant effect on the treatment of colorectal cancer (Wang et al., 2022), which suggests that multi-target therapy may be a potential therapeutic strategy. Traditional Chinese medicine (TCM) is an intricate system with multiple targets (Ma et al., 2015). Different from current perspective of “one target-one drug,” TCM emphasizes the holistic and systematic of whole body. Thanks to the advance of bioinformatics, the fresh network pharmacology is based on public databases and gradually becomes a tool to explore the underlying mechanisms of TCM, from point to network. As an advance technique, the tool updates the research conception and breaks original mode. Besides, it helps to evaluate the reasonableness and toxic side effect of TCM by providing the compound-target-pathway networks. The predictability is a key advantage of network pharmacology, which focuses the co-regulation of different signaling pathways, improves the clinical effect of drugs, decreases the toxic and side effects, and eventually improves the outcome of clinical trials and economizes the cost of drug discovery. Currently, network pharmacology has been widely used in the research of TCM for the cancer therapy (Hong et al., 2017; Wang et al., 2018). Due to network pharmacology has the advantage that well understanding of the principium of systems biology and interactive network, it has attracted a lot of attention, which facilitates the development of novel drug discovery.

Cinobufotalin is an extract derived from the secretions of the traditional Chinese medicine giant toads (Emam et al., 2012). Cinobufotalin was previously mainly used as a cardiotonic, diuretic and a hemostatic agent (Chen et al., 1951). Recent studies have reported that cinobufotalin has antitumor activity and enhances chemotherapeutic effect (Li Y. et al., 2019; Liu et al., 2022). Besides, it has been shown that cinobufotalin induces apoptosis in lymphoma cells (Emam et al., 2012). Cinobufotalin also blocks the growth and metastasis of the tumor by down-regulating the expression of vascular endothelial growth factor (VEGF) and epidermal growth factor receptor (EGFR) (Mao et al., 2020). However, the potential molecular mechanisms of cinobufotalin in COAD are not well-studied.

In this study, we clarified underlying mechanisms of cinobufotalin against COAD using network pharmacology. First, we determined that cinobufotalin has an anti-colon cancer effect. Then we determined potential targets of cinobufotalin and COAD and the share pathways in which those targets play roles. We also checked overlap in those pathways and the networks. In addition, transcriptome sequencing was employed to test the role and mechanism of cinobufotalin on COAD, as analyzed by network pharmacology. Molecular docking results indicated that cinobufotalin interacts with the predicted targets. In a word, our results show that potential mechanisms of cinobufotalin in COAD and provided a theoretical support for cinobufotalin against COAD. More important, this research showed that network pharmacology is a feasible tool to facilitate the development of anticancer drugs.

## Materials and Methods

### Cell Culture and Reagent

The human colon cancer cells HCT8, LoVo and HCT116 were cultured in RPMI1640 containing 10% (v/v) fetal bovine serum (FBS, ExCell Bio, China) and 1% (v/v) penicillin/streptomycin. The cells were cultured at 37°C in an incubator supplied with 5% CO_2_. Cinobufotalin injection (Huachansu Zhusheye) was produced from Anhui China Resources Jinchan Pharmaceutical Co., Ltd.

### Candidate Targets of Cinobufotalin and COAD

The targets of cinobufotalin were predicted from ChEMBL database (https://www.ebi.ac.uk/chembl/), Comparative Toxicogenomics Database (http://ctdbase.org/), PharmMapper Server (http://www.lilab-ecust.cn/pharmmapper/) (Wan et al., 2019), Swiss Institute of Bioinformatics (https://www.expasy.org/) and HERB database (http://herb.ac.cn/). After removing duplicates, a total of 390 candidate targets of cinobufotalin was collected. The known therapeutic targets of drugs used in the treatment of COAD were acquired from DisGeNET database (http://www.disgenet.org/) (Piñero et al., 2020), Phenopedia (https://phgkb.cdc.gov/PHGKB/startPagePhenoPedia.action) (Yu et al., 2010), KEGG disease database (https://www.kegg.jp/kegg/disease/), GeneCards databases (http://www.genecards. org/) and MalaCards databases (https://www.malacards.org/). After removing duplicates, a total of 7,433 targets related to COAD were collected and used for data analysis.

### Network Construction

STRING online database (https://string-db.org/) was applied to obtain the protein-protein interaction network (PPI) data of the molecular targets of cinobufotalin and COAD, where the parameter organism was set to *Homo sapiens*, confidence is high confidence (0.900), and other basic settings were the default value. Cytoscape 3.9.1 (http://www.cytoscape.org) (Shannon et al., 2003) was employed to establish the PPI relationship network and perform topological analysis. To further characterize the molecular mechanism of cinobufotalin on COAD, the drug-target-pathway networks was generated using Cytoscape. In these graphical networks, the compounds, proteins, or pathways were expressed as nodes, whereas the compound-target or target pathway interactions were expressed as edges. The “degree” is an important parameter for the network pharmacology approach, which represents the number of related nodes to a particular node in the network. The greater the degree of a node, the more biologically important it is. Therefore, the top ingredients and targets were screened out by the Network Analyzer in Cytoscape based on the major parameter of “degree”.

### Pathway Enrichment Analysis

Metascape (https://metascape.org/gp/index.html) is a powerful online tool for analyzing gene function annotation and provides gene enrichment analysis (Zhou et al., 2019). This tool was used to analyze the gene ontology (GO), the Kyoto Encyclopedia of Genes and Genomes (KEGG) and Reactome pathway enrichment analysis.

### Transcriptome Sequencing

Total RNA was extracted using Trizol reagent (Thermo Fisher, 15596018) following the manufacturer’s procedure. The total RNA quantity and purity were analysis of Bioanalyzer 2,100 and RNA 6000 Nano LabChip Kit (Agilent, CA, USA, 5,067-1,511), high-quality RNA samples with RIN number >7.0 were used to construct sequencing library. After total RNA was extracted, mRNA was purified from total RNA (5ug) using Dynabeads Oligo (dT) (Thermo Fisher, CA, USA) with two rounds of purification. Following purification, the mRNA was fragmented into short fragments using divalent cations under elevated temperature (Magnesium RNA Fragmentation Module (NEB, cat. e6150, USA) under 94°C 5–7min). Then the cleaved RNA fragments were reverse-transcribed to create the cDNA by SuperScript™ II Reverse Transcriptase (Invitrogen, cat.1896649, USA), which were next used to synthesise U-labeled second-stranded DNAs with E. coli DNA polymerase I (NEB, cat. m0209, USA), RNase H (NEB, cat. m0297, USA) and dUTP Solution (Thermo Fisher, cat. R0133, USA). An A-base was then added to the blunt ends of each strand, preparing them for ligation to the indexed adapters. Each adapter contained a T-base overhang for ligating the adapter to the A-tailed fragmented DNA. Dual-index adapters were ligated to the fragments, and size selection was performed with AMPureXP beads. After the heat-labile UDG enzyme (NEB, cat. m0280, USA) treatment of the U-labeled second-stranded DNAs, the ligated products were amplified with PCR by the following conditions: initial denaturation at 95°C for 3 min; 8 cycles of denaturation at 98°C for 15 s, annealing at 60°C for 15 s, and extension at 72°C for 30 s; and then final extension at 72°C for 5 min. The average insert size for the final cDNA librarys were 300 ± 50 bp. At last, we performed the 2 × 150 bp paired-end sequencing (PE150) on an Illumina Novaseq™ 6,000 (LC-Bio Technology CO., Ltd., Hangzhou, China) following the vendor’s recommended protocol.

### Gene Set Enrichment Analysis

Gene set enrichment analysis was performed using GSEA 4.0.3 (http://software.broadinstitute.org/gsea/index.jsp) in which the hallmark gene set “c5. go.v7.4. symbols.gmt” and “c2. cp.kegg.v7.5. symbols.gmt” were adopted.

### Molecular Docking

In order to test the reliability of the top 4 targets-cinobufotalin interactions and explore the accurate binding modes, we performed molecular docking analysis by using the Discovery Studio (DS) v3.5 (Biovia Inc. San Diego, CA, USA). The crystal structures of proteins (targets) were extracted from Protein Data Bank (https://www.rcsb.org/). The SDF format files of the 3D structure of cinobufotalin were downloaded from the PubChem database (https://pubchem.ncbi.nlm.nih.gov/).

### Immunohistochemical Analysis

The expression of SRC, PIK3R1, MAPK1 and PIK3CA by immunohistochemistry (IHC) comes from the HPA database (https://www.proteinatlas.org/) (Uhlén et al., 2015).

### Statistics

Data were analyzed using GraphPad Prism 8 (GraphPad Software Inc., San Diego, CA, USA). All data are presented as mean ± standard deviation. Comparison of two groups was conducted using the two-tailed Student’s t-test. A value of *p* < 0.05 was considered to indicate a statistically significant difference.

## Results

**FIGURE 1:**
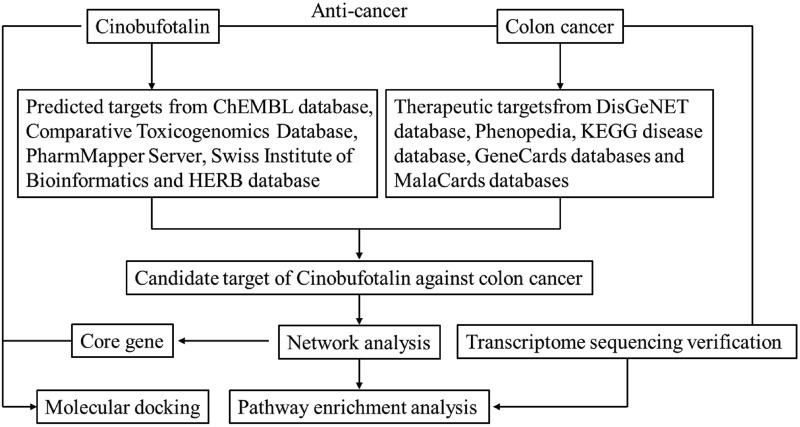
Workflow of the systematic strategies to explore the mechanisms of cinobufotalin against colon cancer.

A bioinformatics diagram through network pharmacology approach that was used to explore the mechanism about cinobufotalin against COAD is shown in Figure 1.

### Cinobufotalin Inhibits Colon Cancer Cell Growth and Proliferation

**FIGURE 2:**
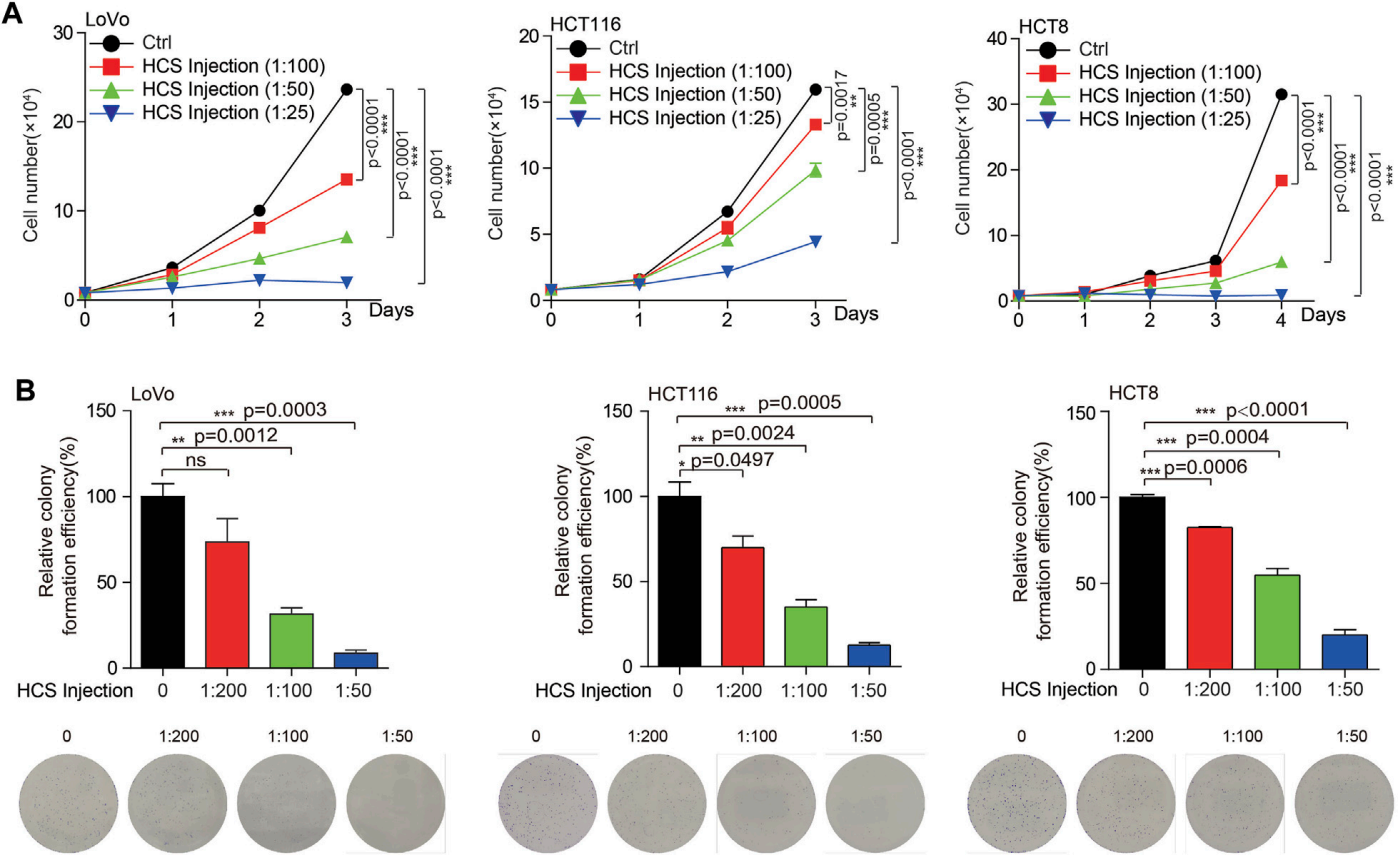
Cinobufotalin inhibits the growth of colon cancer cells **(A)** Cell growth was determined by cell counting in colon cancer cells (LoVo, HCT116 and HCT8) treated with gradient concentration of cinobufotalin **(B)** Cell proliferation was determined by colony formation in colon cancer cells (LoVo, HCT116 and HCT8) treated with gradient concentration of cinobufotalin. Error bars represent mean values ±SD from three replicates of each sample (ns: *p* > 0.05; *: 0.01 < *p* < 0.05; **: 0.001 < *p* < 0.01; ***: *p* < 0.001).

Currently, the clinical treatment of colon cancer is not satisfactory due to the existence of drug resistance. Our previous work found that targeting multi-targets significantly inhibited the growth of colorectal cancer (Wang et al., 2022). Therefore, multi-target therapy may be a promising strategy for colon cancer treatment. TCM is a complex system with multiple components, which also means multiple potential targets. For the reason that we intend to search for possible Chinese medicines for the treatment of colon cancer. Cinobufotalin is a TCM derived from animals, which has the functions of detoxification, swelling and pain relief. In addition, cinobufotalin has also been found to have antitumor activity in cancers, such as liver and lung cancer (Han et al., 2021; Li et al., 2022). However, the role of cinobufotalin in colon cancer has not been explained. To this end, when treated colon cancer cells with cinobufotalin, we found that the COAD cell growth and proliferation were significantly inhibited in a dose dependent manner (Figure 2). This result indicates that cinobufotalin has an anti-colon cancer function, which also lays the foundation for our subsequent network pharmacology analysis.

### Targets of Cinobufotalin Against COAD

**FIGURE 3:**
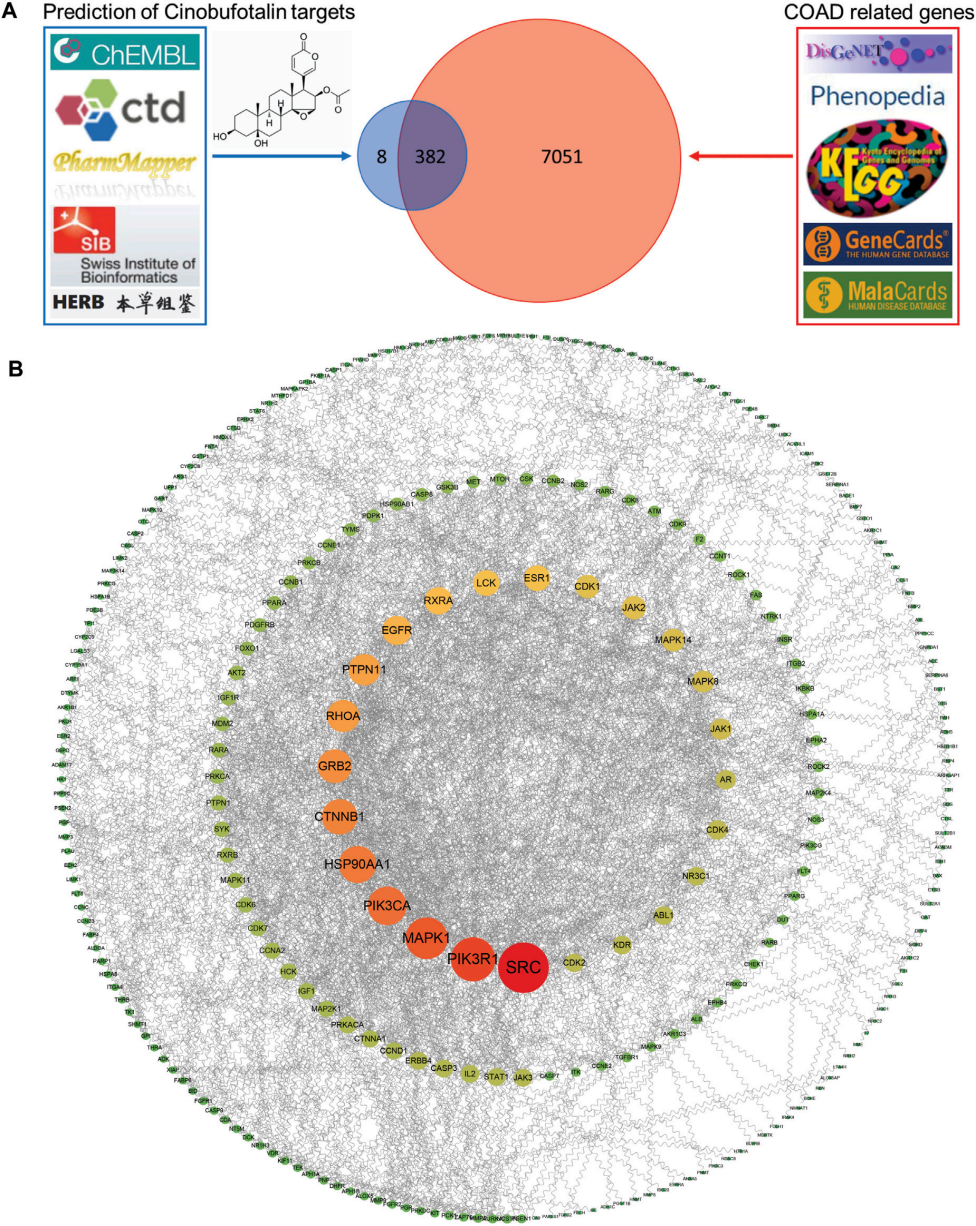
Identification of the drug-target interactions **(A)** Identification of the targets by taking an intersection of cinobufotalin target genes and COAD-related genes from different public database as indicated **(B)** The PPI network was constructed for the shared candidate targets of cinobufotalin and COAD using Cytoscape.

To determine the mechanism of cinobufotalin in anti-colon cancer, we first integrated the targets of cinobufotalin and COAD. The five databases (DisGeNET database, Phenopedia, KEGG disease database, GeneCards databases, and MalaCards databases) were investigated, collecting a total of 7,433 COAD target genes after removing duplicates. Meanwhile, targets for cinobufotalin were collected from the five databases (ChEMBL database, Comparative Toxicogenomics Database, PharmMapper Server, Swiss Institute of Bioinformatics and HERB database). The 390 target genes were yielded after removing duplicates. The target genes of COAD and cinobufotalin were intersected to generate 382 potential targets (Figure 3A). Immediately after, the 382 target genes were imported into the STRING database to generate the PPI network map. In order to construct the interaction network between proteins and unearth the core regulatory genes, the PPI network of the candidate targets was conducted (Figure 3B). The top 10 targets of the PPI network results were determined and ranked by degree. These genes include SRC, PI3K3R1, MAPK1, PIK3CA, HSP90AA1, CTNNB1, GRB2, RHOA, PTPN11, and EGFR.

### The Pathway Enrichment Analysis of Cinobufotalin Against COAD

**FIGURE 4:**
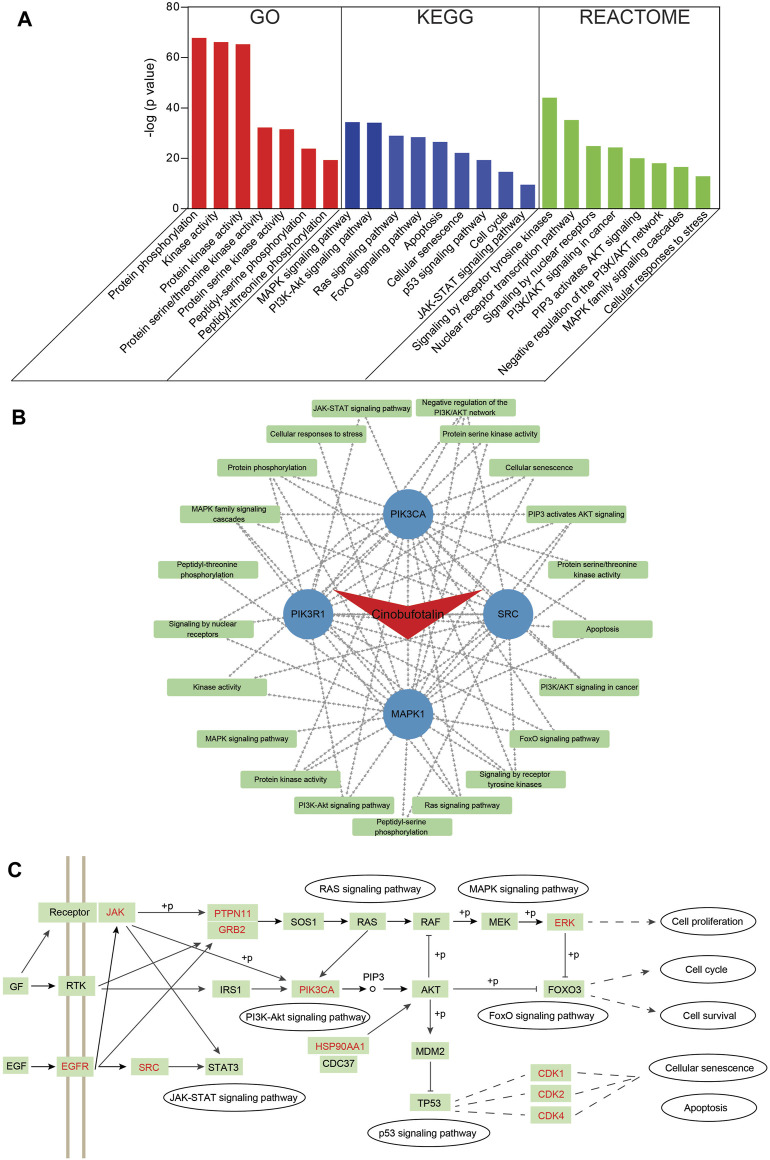
Functional characterization of cinobufotalin against COAD intersecting genes **(A)** GO, KEGG and Reactome enrichment analysis was conducted for intersecting genes using Metascape tools **(B)** The drug-target protein-pathway network was constructed using Cytoscape **(C)** The integrated pathways map of cinobufotalin against COAD. “+p” stands for phosphorylation modification. The targets marked in red are the predicted targets of cinobufotalin.

To identify pathways involved in overlapping genes, pathway enrichment analysis was performed. First, the GO enrichment analysis was examined to explore biological functions of cinobufotalin in COAD. The top seven enriched biological functions were chosen for the display of histograms (Figure 4A). The biological functions of these genes mainly include protein phosphorylation, kinase activity, protein kinase activity, protein serine/threonine kinase activity, and protein serine kinase activity. This results suggest that cinobufotalin mainly participated in the treatment of COAD by mediating protein phosphorylation process that contribute to the understanding of the signal pathway mechanisms in COAD.

To get an in-depth understanding of the role of cinobufotalin against COAD, the 184 signaling pathways were found by the KEGG pathway enrichment analysis. The top nine vital signaling pathways are displayed in histograms. As shown in Figure 4A, many signaling pathways are closely related with COAD, such as MAPK, PI3K-AKT, Ras, FoxO, p53, JAK-STAT signaling pathway, cell cycle, cellular senescence, and apoptosis. This is also in line with the function of cinobufotalin to regulate protein phosphorylation, which ensures the signal transmission by phosphorylating target proteins. Besides, we also analyzed from Reactome enrichment analysis, and found that the signaling pathway similar to KEGG (Figure 4A). These data suggest that cinobufotalin alters protein phosphorylation modifications during signaling in colon cancer.

To further explore the association of targets and pathways, we enter the results of enriched pathway and core target into the Cytoscape software to obtain a cinobufotalin-target-pathway network (Figure 4B). The 26 nodes and 67 lines were showed in the cinobufotalin-target-pathway network. The results found that the top four targets, namely, SRC, PIK3R1, MAPK1, and PIK3CA, have a highest degree in this list, which implies their importance. Importantly, we integrated the signaling pathways involved in cinobufotalin in colon cancer and found that these pathways are interconnected (Figure 4C). And the targets we identified occupied key positions, suggesting that cinobufotalin interacts with them to regulate the signal transduction process. Based on the above signal pathway analysis results, it was shown that cinobufotalin regulates protein phosphorylation in the signal transduction pathway, which in turn affects cell apoptosis, senescence, and cell cycle processes.

### Transcriptome Sequencing Analysis in COAD Cells Treated With Cinobufotalin

**FIGURE 5:**
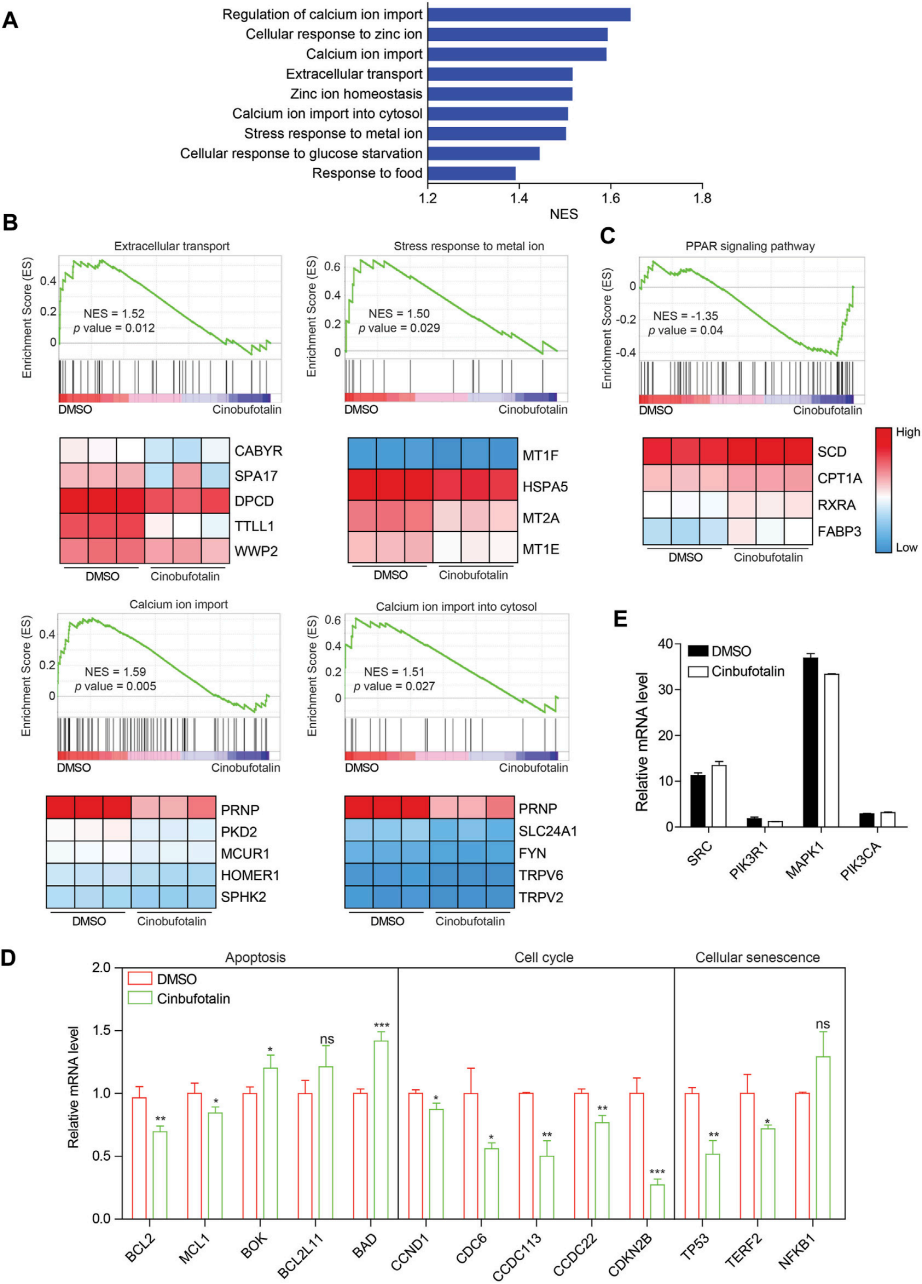
Cinobufotalin inhibits the extracellular signal response of tumor cells **(A)** The core-enriched signaling pathways in cinobufotalin treatment or control groups. NES, normalized enrichment score **(B,C)** GSEA pathway enrichment analyses of HCT8 cells with the treatment of cinobufotalin. Genes with significantly different expression are shown as heat maps **(D)** The expression of apoptosis, cell cycle and cellular senescence related genes in HCT8 cells with the treatment of cinobufotalin **(E)** The expression of hub genes in HCT8 cells with the treatment of cinobufotalin. Error bars represent mean values ±SD from three replicates of each sample (ns: *p* > 0.05; *: 0.01 < *p* < 0.05; **: 0.001 < *p* < 0.01; ***: *p* < 0.001).

To validate the regulation of cinobufotalin against COAD as analyzed by network pharmacology, transcriptome sequencing were conducted. Gene set enrichment analysis (GSEA) of pathway enrichment showed that extracellular stimulus response was inhibited after the treatment of cinobufotalin (Figure 5A), which implies that cinobufotalin block the cell communication with the outside world. Among them, the stimulation of metal ions is the most obvious. Further analysis found that cinobufotalin antagonized the import of extracellular calcium ions (Ca^2+^) and down-regulated the expression of genes related to calcium ion import (Figure 5B). The above result is surprising, as Emam et al. reported that cinobufotalin increased the intracellular Ca^2+^ concentration (Emam et al., 2012). Besides, it was reported that cinobufotalin also inhibits the Na^+^-K^+^ pump and inhibit the transport of Na^+^ and K^+^ (Akimova et al., 2005). These results suggest that cinobufotalin alters the cell’s response to metal ions.

As the second messenger, Ca^2+^ plays an vital role in regulating biological processes such as cell growth, cell death and immunomodulatory (Marchi et al., 2020). In addition, calcium ions are also involved in multiple signal transduction pathways (Marchi et al., 2020). Therefore, decreased calcium import also represents a reduced ability of cells to transduce extracellular signals. Not only that, we also found that the PPAR signaling pathway was activated by cinobufotalin (Figure 5C). The PPAR signaling pathway is reported to be inhibited by the MAPK pathway (Papageorgiou et al., 2007), which also partly indicates that cinobufotalin inhibits the MAPK signaling pathway. In addition, we also analyzed genes related to apoptosis, cell cycle, and cellular senescence. The results showed that the expression of anti-apoptotic genes was decreased and the expression of pro-apoptotic genes was up-regulated (Figure 5D), suggesting the occurrence of apoptosis. In addition, the expression changes of cell cycle and cell senescence-related genes indicated that cinobufotalin induces cell senescence and inhibits cell cycle. Collectively, the results of transcriptome sequencing verified the analysis results of network pharmacology, and cinobufotalin impedes the signal exchange between tumor cells and the outside world, promotes apoptosis and cell senescence, and ultimately inhibits the progression of colon cancer.

### Binding Capacity Between Cinobufotalin and Target by Molecular Docking

**FIGURE 6:**
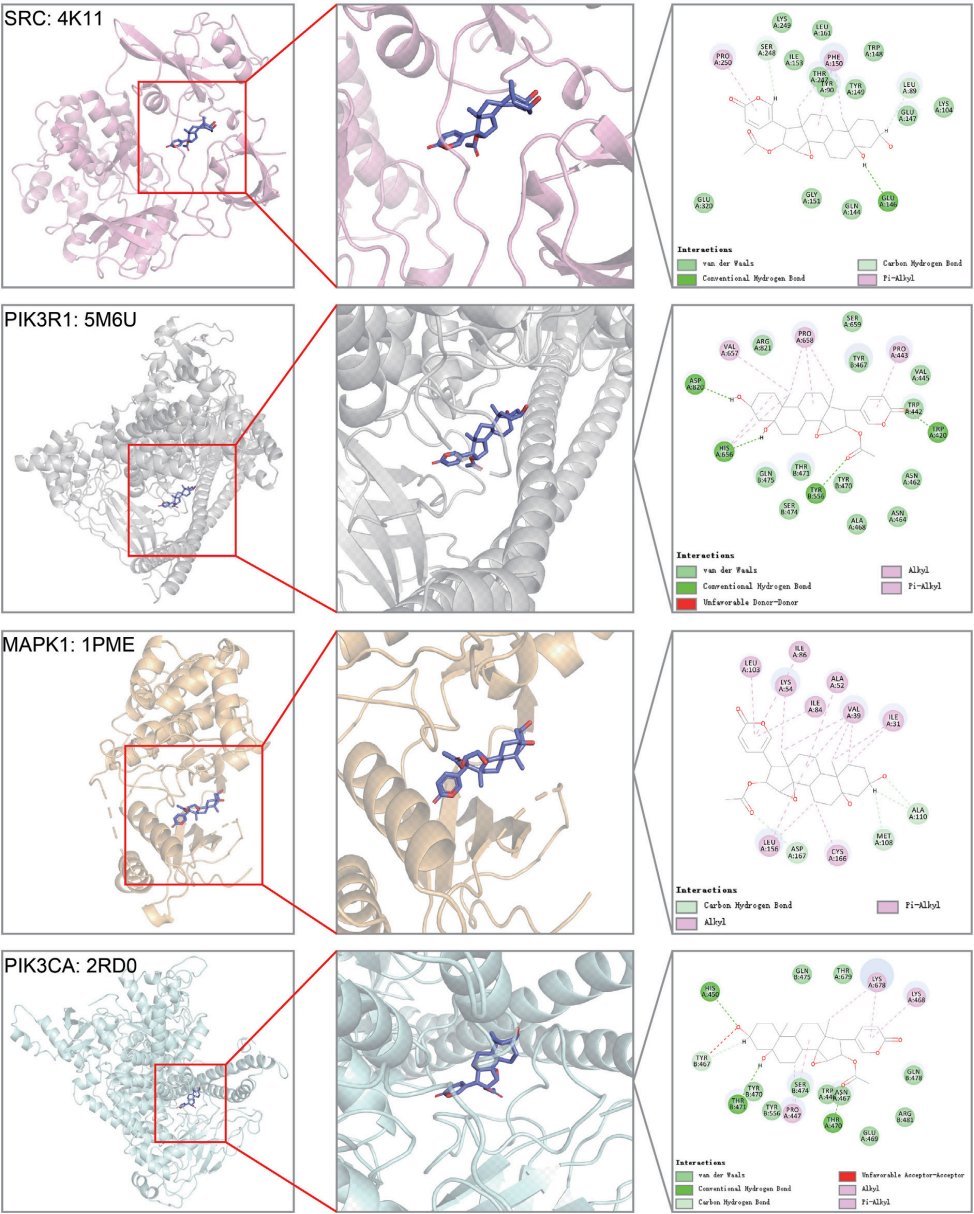
Molecular models of cinobufotalin binding to predicted protein targets. Proteins (SRC = 4K11, PIK3R1 = 5M6U, MAPK1 = 1PME, and PIK3CA = 2RD0) interact with a cinobufotalin molecule, and different proteins are distinguished by different colors.

Among the top genes in the cinobufotalin-target-pathway network, SRC, PIK3R1, MAPK1, and PIK3CA were also considered to be highly related to COAD. However, in the sequencing results, we found that cinobufotalin did not significantly change the expression of core genes (Figure 5E), so we assumed that cinobufotalin and the target protein act directly. Molecular docking is used to simulate the interaction between small molecular drugs and receptor proteins, and then designs and optimizes drugs. It is an useful tool in the field of computer-aided drug design (Pinzi and Rastelli, 2019). Therefore, protein 4K11 encoded by SRC, protein 5M6U encoded by PIK3R1, protein 1PME encoded by MAPK1, and protein 2RD0 encoded by PIK3CA were chosen to run molecular docking (Figure 6). Cinobufotalin could easily enter and bind the active pocket of these five proteins. The above results demonstrate that cinobufotalin functions by directly interacting with target proteins, rather than regulating their expression abundance. For kinase proteins, their function depends mainly on activity, rather than expression abundance. Therefore, combining with cinobufotalin significantly changes its activity and cause functional dysregulation.

**FIGURE 7:**
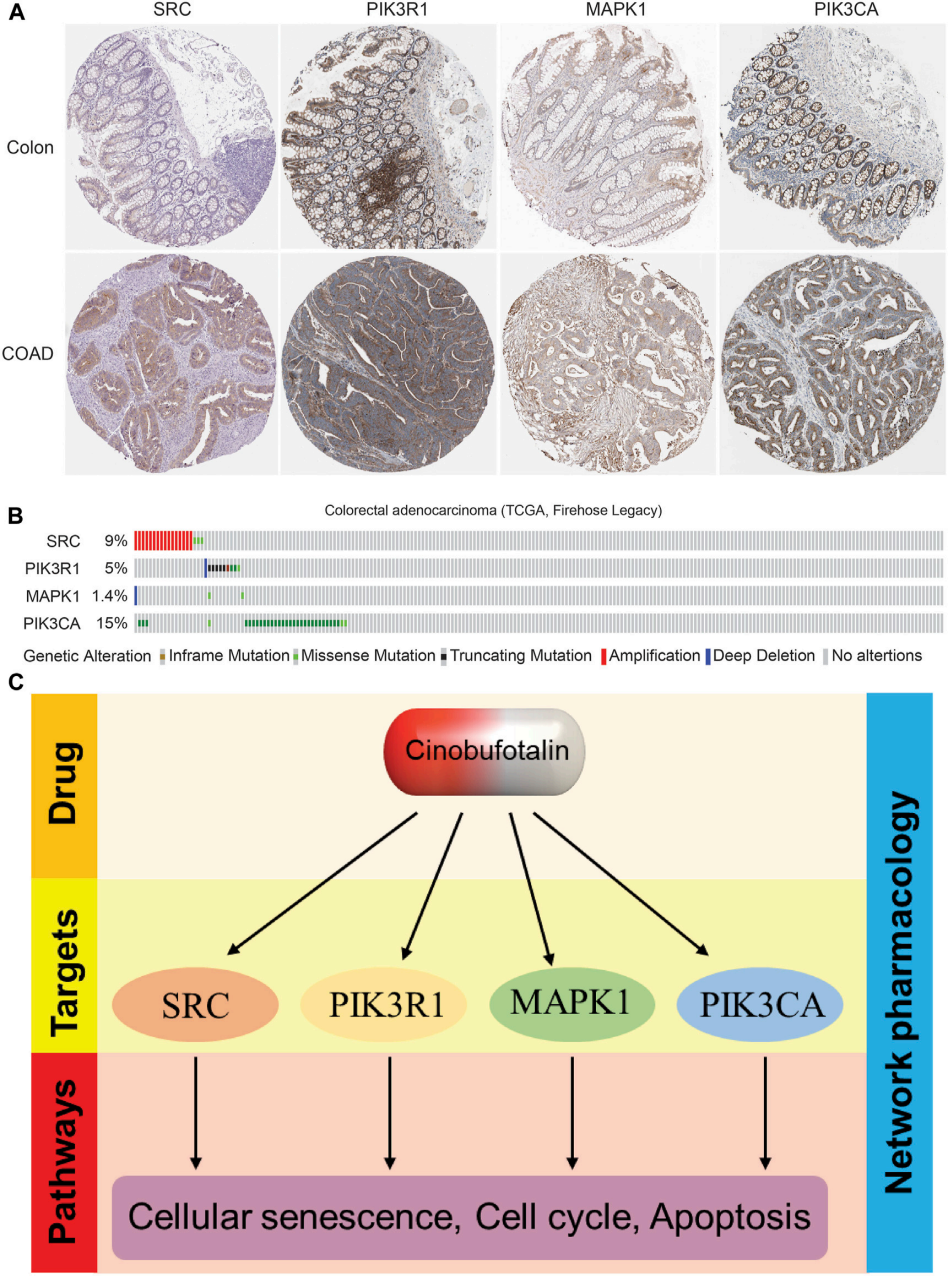
The expression and mutation frequency of key targets in colon cancer **(A)** Representative IHC staining of SRC, PIK3R1, MAPK1 and PIK3CA in COAD from HPA database **(B)** The types and frequency of mutations of SRC, PIK3R1, MAPK1 and PIK3CA in COAD patients **(C)** Proposed model: The role and mechanism of cinobufotalin against COAD.

To further prove our conclusions, we analyzed the expression abundance of target proteins in COAD based on HPA database, and the results showed that the expression was significantly elevated in COAD (Figure 7A), which suggests that these kinases play a tumor-promoting role in tumor tissues. Interestingly, we also found that these kinase proteins also have a higher mutation frequency in COAD (Figure 7B), suggesting that their activity also plays an important role in tumor progression. In this study, cinobufotalin did not change the expression, but only exerted a tumor suppressor function by inhibiting its activity, which may prove that cinobufotalin is an enzyme activity inhibitor.

## Discussion

With the improvement of living standards, the incidence of colon cancer is increasing. Although the treatment of cancer is also improving, it lags behind the development of cancer, so the survival rate of cancer patients has not improved significantly. The main clinical treatment methods for colon cancer are surgical resection and radiotherapy and chemotherapy. However, surgical resection is suitable for the early stage of the tumor, and radiotherapy and chemotherapy will produce obvious side effects and increase the suffering of patients. Therefore, developing new therapeutic strategies is an urgent task in the treatment of colon cancer. In previous studies, we found that the multi-targets for the treatment of colorectal cancer have a visible effect (Wang et al., 2022). Therefore, multi-targeted tumor therapy may be a promising therapeutic strategy.

TCM has received extensive attention owing to its treatment effect in anti-tumor treatment (Yuan et al., 2017; Man et al., 2021). Growing evidence that TCM could also improve the effectiveness of chemotherapy drugs in treating cancer (Zhou et al., 2016; Yuan et al., 2017). Therefore, we must acknowledge that TCM is a key source for the discovery of new anti-colon cancer drugs. Cinobufotalin is the main component of cinobufotalin injection (Li et al., 2021). Cinobufotalin was previously found to inhibit the activity of Na^+^/K^+^-ATP enzyme (Akimova et al., 2005). In addition, cinobufotalin has inhibitory effects on many tumors, including lung, liver, ovarian, breast, and gastric cancers (Kai et al., 2014; Cheng et al., 2015; Afroze et al., 2018; Li J. et al., 2019; Sun et al., 2019). More importantly, cinobufotalin also alters the sensitivity of chemotherapeutic drugs (Li Y. et al., 2019; Liu et al., 2022). Therefore, the anticancer effects of cinobufotalin have attracted much attention.

Based on the theory of systems biology, network pharmacology integrates pharmacology, public databases, and bioinformatics, emphasizing the coordinated regulation of signalling pathways. Here, the underlying therapeutic target of cinobufotalin was identified by network pharmacology, and the mechanism and role of cinobufotalin against colon cancer was expounded. After collecting and analysing the target genes of cinobufotalin and colon cancer, it was found that cinobufotalin may affect the phosphorylation of proteins to regulate tumor cell apoptosis, cell cycle and cell senescence. Phosphorylation modification of proteins is an important event in maintaining cell life activities, such as participating in signal transduction processes. In the following pathway enrichment analysis, we found that several canonical extracellular signalling pathways were enriched, indicating that cinobufotalin inhibits the signal transduction pathway by inhibiting protein phosphorylation. Furthermore, to validate the results drawn from network pharmacology, we used cinobufotalin-treated colon cancer cells for transcriptome sequencing. The analysis found that cinobufotalin inhibits the response of colon cancer cells to extracellular signals, especially calcium ions. It is well known that calcium ion is the second messenger and participates in the process of signal transduction (Marchi et al., 2020). These results again indicate the regulatory effect of cinobufotalin on the signal transduction of tumor cells.

In the pathway enrichment analysis, we found that cinobufotalin inhibits MAPK, PI3K-AKT and JAK-STAT signalling pathway. The mitogen-activated protein kinases/extracellular signal-regulated kinase (MAPK/ERK) pathway includes several signalling components and phosphorylation events that play roles in the occurrence and development of tumor. These activated kinases transmit extracellular signals that regulate cell proliferation, cell death and other functions. Aberration in the MAPK/ERK signalling pathway induces cell migration, senescence, differentiation of cancer (Taupin and Podolsky, 1999; Dong et al., 2002; Hommes et al., 2003; Sun et al., 2015). The alterations of KRAS and BRAF frequently occur in COAD leading to MAPK dysfunction (Fang and Richardson, 2005). Mutated RAS has oncogenic capacity, alters downstream molecules and interferes with transcription factor expression (Wan et al., 2019). The key driver of COAD development is aberrant expression of RAS proteins (Campbell et al., 2007). Therefore, cinobufotalin inhibits the MAPK signalling pathway to inhibit colon cancer progression. Similarly, high expression of the PI3K/AKT/mTOR signalling has been found in several cancers, especially in COAD (Fresno Vara et al., 2004). Due to the vital roles in colorectal cancer progression, they are considered a compelling therapeutic target. Besides, the JAK/STAT signal identified as a biomarker of COAD for the therapy (Tang et al., 2019). Not only that, we also identified EGFR and its signalling pathway, and anti-EGFR drugs (cetuximab or panitumumab) are commonly used clinically for the treatment of colorectal cancer (Dekker et al., 2019). It is worth noting that these signalling pathways are not independent of each other, but are interrelated (Figure 3C). The core genes identified by network pharmacology play a role in mutual regulation. Molecular docking result shows that cinobufotalin directly interact with these key proteins. In conclusion, through network pharmacology analysis and transcriptome sequencing verification, we found that cinobufotalin inhibits extracellular signal transduction to cut off the communication between cells and the outside, inhibit tumor cell cycle, and promote cell senescence and apoptosis (Figure 7C).

## Conclusion

Take advantage of network pharmacology, we understood the potential anti-COAD mechanism of cinobufotalin, using bioinformatics methods to generate potential targets of cinobufotalin in COAD. SRC, PIK3R1, MAPK1, and PIK3CA are considered to be core genes. Pathway enrichment results indicated that the mechanism of cinobufotalin against COAD is to inhibit extracellular signal transduction, promote tumor cell apoptosis, and regulate tumor cell cycle. However, further experiments are needed to prove these predictions. At last, we hope that identified targets and mechanisms could be verified in *vitro* and *in vivo* experiments in the future.

## Data Availability

The original contributions presented in the study are included in the article/supplementary material, further inquiries can be directed to the corresponding authors.
